# Proteomic analysis of oat (***Avena sativa*** L.) under drought stress using tandem mass tag labeling

**DOI:** 10.1371/journal.pone.0322022

**Published:** 2025-04-29

**Authors:** Caijin Chen, Mingfang Bao, Yanxia Zeng, Xuemin Wang, Wenhui Liu

**Affiliations:** 1 Qinghai Academy of Animal Science and Veterinary Medicine, Qinghai University, Xining, Qinghai, China; 2 Guyuan Branch, Ningxia Academy of Agricultural and Forestry Sciences, Guyuan, Ningxia, China; 3 Institute of Animal Sciences, Chinese Academy of Agricultural Sciences, Beijing, China; United Arab Emirates University, UNITED ARAB EMIRATES

## Abstract

Drought is a major abiotic stress that limits oat growth. This study investigated the phenotypic, physiological, and proteomic differences between drought-resistant (Grain King [G]) and drought-susceptible (XiYue [X]) oat varieties under drought stress (soil water content of 15% ± 5% of field water-holding capacity) and normal conditions (soil water content of 75% ± 5% of field water-holding capacity). Phenotypic analysis showed that plant height, aboveground biomass, and underground biomass decreased under drought stress in both varieties, with variety X exhibiting a greater reduction. Physiological analysis revealed increased malondialdehyde content, soluble sugar (SS) content, and superoxide dismutase (SOD) and peroxidase (POD) activities in both varieties under drought stress, though variety X showed smaller increases. Proteomic analysis identified 151 differentially accumulated proteins (DAPs) in variety G and 792 in variety X. Further analyses showed that the DAPs in variety G, which were highly correlated with POD and SOD activities and SS content, were primarily involved in energy metabolism, protein translation, RNA processing, amino acid metabolism, and protein folding. Conversely, in variety X, the DAPs were mainly associated with RNA processing, protein stabilization, plant photosynthesis, intracellular signal transduction, and protein folding. Further analysis suggested that variety G significantly upregulated proteases related to photosynthesis, catalysts involved in citrulline synthesis, temperature-induced lipid transport proteins, fibrillin proteins linked to stress tolerance signal transduction and response, and shearing factors involved in mRNA shearing—proteins that were not significantly upregulated in variety X. These proteins may play essential roles in protecting oats from drought stress. Overall, this research elucidates the drought resistance mechanisms of different oat varieties at the protein level.

## Introduction

Drought is a prominent threat to agriculture worldwide [1]. When crops experience prolonged water deficits, they evolve complex mechanisms to sense water availability in their environment and activate stress responses at the morphological, physiological, biochemical, and molecular levels, reprogramming their metabolism and growth to establish new homeostasis [2–4]. Morphologically, most plants respond to drought stress through adaptive changes in root, stem, and leaf morphology; leaf orientation; stomatal closure; root system deepening; reduced branch growth; and leaf senescence and dormancy [3–6]. Physiologically and biochemically, drought stress induces the production and accumulation of organic solutes, including proline, betaine, sugar, and sugar alcohols, which prevent membrane disintegration and enzyme inactivation [7–9]. At the molecular level, drought stress activates multiple drought-related functional genes (e.g., *GhNAC4*, *ZmGA20ox3*, and *TaWRKY31*) and induces the synthesis and expression of stress effectors and regulatory proteins involved in transcription, reactive oxygen species scavenging, antioxidant regulation, and signal transduction pathway activation [10–14]. These mechanisms enable plants to enhance their ability to avoid damage (stress avoidance mechanisms) or maintain metabolic functions under drought conditions (stress tolerance mechanisms) [15].

Oat (*Avena sativa* L.) is one of the six most grown cereal crops worldwide, with over 10 million hectares under cultivation; it is primarily used as food, feed, and forage [15,16]. Oat contains highly valuable compounds for industrial applications, including glucan, oil, and protein [17,18]. In China, policies such as “grain to feed” and the rapid development of animal husbandry have significantly increased the demand for high-quality forage oats, leading to expanded planting areas. However, drought has severely impacted oat productivity, and it has been shown that oats are predominantly grown in drought-prone or low-nutritional areas that are unsuitable for major food crop production in most countries [19]. Therefore, it is crucial to conduct research on drought-related genes and proteins in oats to analyze their drought resistance mechanisms at the molecular level, better guide the selection and breeding of drought-resistant varieties, and improve their production.

Proteomics involves the study of functions, recognition, and regulation of proteins in cells, subcellular compartments, and tissues. Numerous proteomic studies have elucidated the effects of drought stress on plant growth and development, as well as the protein expression strategies employed by various plants in response to drought. For instance, drought stress decreases rubisco-binding protein levels and inhibits photosynthesis in alfalfa leaves [20]. In maize, drought upregulates protective and stress-related proteins, primarily chaperone proteins and dehydrins, and causes one-to-one changes in antioxidant enzyme activities and detoxification protein levels [21]. Similarly, drought stress reduces photosynthesis, carbohydrate levels, protein levels, and defense- and energy metabolism-related protein levels in a drought-tolerant safflower variety, with these parameters recovering upon rehydration; however, no significant differences are observed in a drought-sensitive variety under drought and rehydration conditions [22]. In soybean roots, proteins involved in osmoregulation, defense signaling, and programmed cell death, expressed in response to short-term drought stress, are critical for drought adaptation [23]. Additionally, under mild drought stress, osmolyte accumulation enhances resistance, whereas under moderate and severe drought stress, unsaturated fatty acid oxidation and glucose and galactose accumulation increase, accompanied by suppression of β-coumarin synthesis and the terpene precursor 2,3-epoxide. Triterpene (glycyrrhetinic acid) accumulation is also affected [24]. Thus, proteomic studies are essential for understanding the complex regulatory mechanisms underlying plant drought resistance. In this study, tandem mass tag (TMT) chemical marker quantitative proteomics technology was used to characterize and quantify proteins in the seedling leaves of various drought-resistant forage oat varieties under drought stress and normal water supply conditions. Differential protein accumulation profiles were screened through comparative analysis to identify key proteins involved in drought stress stimulation and regulatory pathways in forage oats. The findings provide a foundation for further research on the mechanisms of oat response to drought stress.

## Materials and methods

### Plant materials

The materials used in this study were commercial varieties of forage oats *Avena sativa* cv. ‘Grain King’ [G] (Baili International Grass, Beijing Co., Ltd.) and *A. sativa* cv. ‘XiYue’ [X] (Ningxia Daxinong Seed Co.). A previous study determined the phenotypic traits of seven oat varieties under drought stress and normal water supply conditions and screened varieties with different drought tolerance levels; among them, varieties G and X were identified as drought-resistant and drought-susceptible, respectively [25].

### Stress treatment

Two treatments, drought stress and normal water supply (control), were set up for each variety, with three replicates planned for each treatment. Normal irrigation was implemented from sowing to 15 days after seedling emergence, maintaining the soil water content at 75% ± 5% of the field water-holding capacity. Drought stress was applied 15 days after seedling emergence, with the soil water content reduced to 15% ± 5% of the field water-holding capacity. This soil moisture level was sustained for 3 days, after which the relevant indicators were measured. For the control conditions, the soil water content was maintained at 75% ± 5% of the field water-holding capacity until measurements. The drought and control treatments for variety G are indicated as GD and GW, whereas those for variety X are indicated as XD and XW, respectively.

### Plant growth conditions

The drought experiment was conducted in a plant growth room at Qinghai University under controlled environmental conditions: 20/25°C (night/day), 50%–60% relative humidity, 500 μmol m⁻^2^·s⁻^1^ of light intensity, and a photoperiod of 8 h/16 h (night/day). For each variety, 240 seeds of uniform size and fullness were selected. The seeds were sterilized using 5% Na_2_ClO_3_, rinsed three times with distilled water, dried, and sown on non-porous pots (24.5 × 35 × 30 cm) filled with soil. The soil’s basic physicochemical properties are listed in Table 1. Fifteen days after emergence, seedlings were thinned out, leaving 20 healthy plants per pot for the experiments.

**Table 1:** Basic physical and chemical properties of the soils.

Organic matter (g/kg)	Total nitrogen (mg/kg)	Total phosphorus (%)	Total potassium (%)	Hydrolytic nitrogen (mg/kg)	Fast-acting potassium (mg/kg)	Effective phosphorus (mg/kg)	Water-soluble salt Total amount (g/kg)	pH
89.33	3.42 × 10^3^	0.17	3.00	241.08	1147.00	273.74	4.78	7.75

### Morphological and physiological index determination methods

Five seedlings were randomly selected from each pot for phenotypic indicators. The roots were cleaned, and the plant height was measured using a scale. The plants’ aboveground and underground parts were then separated, dried at 80°C ± 2°C until constant weight, and measured.

The malondialdehyde (MDA) content was determined based on a colorimetric technique using thiobarbituric acid, as described by Na Zhang [26]. Additionally, the soluble sugar (SS) content was determined by referring to the anthrone method described by Jun Wang [27]. Furthermore, the total superoxide dismutase (SOD) and peroxidase (POD) activities were determined using the SOD (A001–3–2) and POD (A084–3-1) test kits (Nanjing Jiancheng Institute of Biological Engineering, Nanjing, China) and water-soluble tetrazolium-1 (WST-1) dye with colorimetric methods.

### Statistics and analysis of phenotypic and physiological data

The data were initially processed using Microsoft Excel 2016 and analyzed using SPSS19.0 statistical software for ANOVA (*p* < 0.05). The data were cartographed using OriginPro 2021(v 9.8.0.200) and R28 (v 3.4.3) statistical software.

### Proteomic analysis

#### Sample preparation.

Sample leaves (≥100mg) were first ground to a powder with a small amount of liquid nitrogen, added to 200 µL of lysis buffer (150 mM tris(hydroxymethyl)aminomethane hydrochloride [Tris–HCl], 4% sodium dodecyl sulfate, 100 mM dithiothreitol [DTT], pH 7.8), sonicated, boiled for 5 min, and precipitated with a trichloroacetic acid-acetone solution. After centrifugation at 16,000 ×g (1 min, 4°C), the tubes were washed twice with cold acetone and air dried. Each sample tube was complemented with 150 µL of lysis buffer, sonicated, and centrifuged at 16,000 ×g for 15 min to remove undissolved cell debris. The supernatants were collected for quantification using the bicinchoninic acid protein kit (Bio-Rad, USA).

#### Protein digestion.

Protein digestion was performed according to the method described by Wisniewski et al. [28]. DTT, detergents, and other low-molecular-weight fractions were removed by centrifugal ultrafiltration (Microcon units, 30 kD) using 200 μL of uric acid (UA) buffer (150 mM Tris–HCl, 8 M urea, pH 8.0). Next, 100 μL of 0.05 M iodoacetamide was added to the UA buffer to block the reduced cysteine residues, and samples were incubated in the dark for 20 min. The filters were washed three times with 100 μL of UA buffer and twice with 100 μL of 25 mM NH_4_HCO_3_. Finally, the protein suspension was digested using 4 μg trypsin in 40 μL of 25 mM NH_4_HCO_3_ overnight at 37°C, and the filtrate was obtained. The peptide concentration was determined using the nanodrop OD_280_ method.

#### TMT labeling of peptides and fractionation.

Peptides were labeled with the TMT reagent according to the manufacturer’s instructions (Thermo Fisher Scientific). Each sample (100 μg peptide equivalent) was reacted with one tube of TMT reagent. After dissolving the sample in 100 μL of 0.05 M triethylammonium bicarbonate solution at pH 8.5, the TMT reagent was dissolved in 41 μL of anhydrous acetonitrile. The reaction mixture was incubated for 1 h at room temperature, 8 μL of 5% hydroxylamine was added, and the sample was incubated for 15 min to quench the reaction. The 12 Multiplex-labeled samples were pooled together and lyophilized. The TMT-labeled peptide mixture was separated on a Waters XBridge BEH130 column (C18, 2.1 × 150 mm, 3.5 μm) on an Agilent 1290 high-performance liquid chromatograph operating at 0.3 mL/min. Buffer A included 10 mM ammonium formate, and buffer B comprised 10 mM ammonium formate and 90% acetonitrile. Both buffers were adjusted to pH 10 with ammonium hydroxide. Thirty fractions were collected for each peptide mixture and linked in series to 15 fractions. The fractions were dried and subjected to liquid chromatography-mass spectrometry (LC-MS) analysis.

#### LC-MS analysis.

For each supernatant, 1 μg of each fraction was analyzed using a nanoliter flow rate Easy nLC1200 chromatography system (Thermo Scientific). The buffers used in the analysis were solution A (0.1% formic acid aqueous solution) and solution B (0.1% formic acid, acetonitrile and water mixture [with 95% acetonitrile]). The column was first equilibrated using 100% of liquid A. The sample was loaded onto a Trap Column (100 µm × 20 mm, C18, 5 µm, Dr. Maisch GmbH) and passed through the analytical column (75 µm × 150 mm, C18, 3 µm, Dr. Maisch GmbH) for gradient separation at a flow rate of 300 nL/min. The liquid phase separation gradient settings were as follows: 0–2 min, liquid B linear gradient from 2%–8%; 2–71 min, liquid B linear gradient from 8%–28%; 71–79 min, liquid B linear gradient from 28%–40%; 79–81 min, liquid B linear gradient from 40%–100%; 81–90 min, liquid B at 100%. The peptides were analyzed via data-dependent acquisition-mass spectrometry using a Q-Exactive HF-X mass spectrometer (Thermo Scientific) after separation. The analysis time was 90 min, the detection mode was positive-ion, the parent ion scan range was 400–1800 m/z, and the primary mass spectrometry resolution settings were as follows: 60,000 @m/z 200; automatic gain control (AGC) target, 3e6; and primary maximum injection time, 50 ms. For the peptide segments’ secondary mass spectrometry, 20 secondary mass spectra of the highest intensity parent ions were triggered after each full scan with the following secondary mass resolution: 45,000 @ m/z 200; AGC target, 1e5; secondary maximum injection time, 50 ms; MS2 activation type, high-energy collisional dissociation; isolation window, 1.2 m/z; and normalized collision energy, 32.

#### Database query and analysis.

The obtained LC-MS/MS raw files were imported into the search engine in Thermo Scientific Proteome Discoverer software (v2.4, Thermo Scientific) for database search. Because no oat-specific database is available, we used the Uniprot-Pooideae database [147368]_924681_20211014.fasta (download date: October 14, 2023; available on https://www.uniprot.org/taxonomy/147368) Protein Data Bank with the 924681 protein sequence. An initial search was set in a precursor to the 6 ppm mass window. The study followed the rules of enzymatic cleavage of trypsin/P, allowing a fragment ion mass tolerance value of 20 ppm and two maximum missing cleavage sites with the following modification sets: fixed modifications, carbamoylmethyl (C), TMT16plex (K), TMT16plex (N term); variable modifications, oxidation (M), acetyl (Protein N term). Peptides require at least six amino acids and one unique peptide per protein. For peptide and protein identification, the false discovery rate (FDR) was set to 1%. The mass spectrometry data for all proteins can be obtained from the iProX database website (IPX0010483000/PXD058831). The TMT reporter ion intensity was used for quantification, and the relative quantification of the sample proteins was performed using the MaxQuant algorithm (http://www.maxquant.org) [29].

### Bioinformatics analysis

Bioinformatics data were analyzed using Perseus44 (v1.6.5.0) software, Microsoft Excel 2016, and R28 (v 3.4.3) statistical software. DAPs were screened with the cutoff of a ratio fold-change of > 1.20 or < 0.83 and *p* < 0.05. Additionally, proteins with significant variance features were identified using hierarchical clustering (Euclidean distance) and analysis of variance (ANOVA). For the enrichment analysis of the specified protein functions, annotation information was extracted from the UniProtKB/Swiss-Prot, Kyoto Encyclopedia of Genes and Genome (KEGG), and Gene Ontology (GO) databases. GO and KEGG enrichment analyses were conducted using Fisher’s exact test, and FDR correction for multiple testing was also performed. GO terms were grouped into three categories: biological process (BP), molecular function (MF), and cellular component (CC). Correlations between sample features and protein expression changes were analyzed using weighted gene co-expression network analysis (WGCNA), and the expression data were clustered hierarchically based on the protein level.

## Results

### Effects of drought stress on oat growth

**Fig 1:**
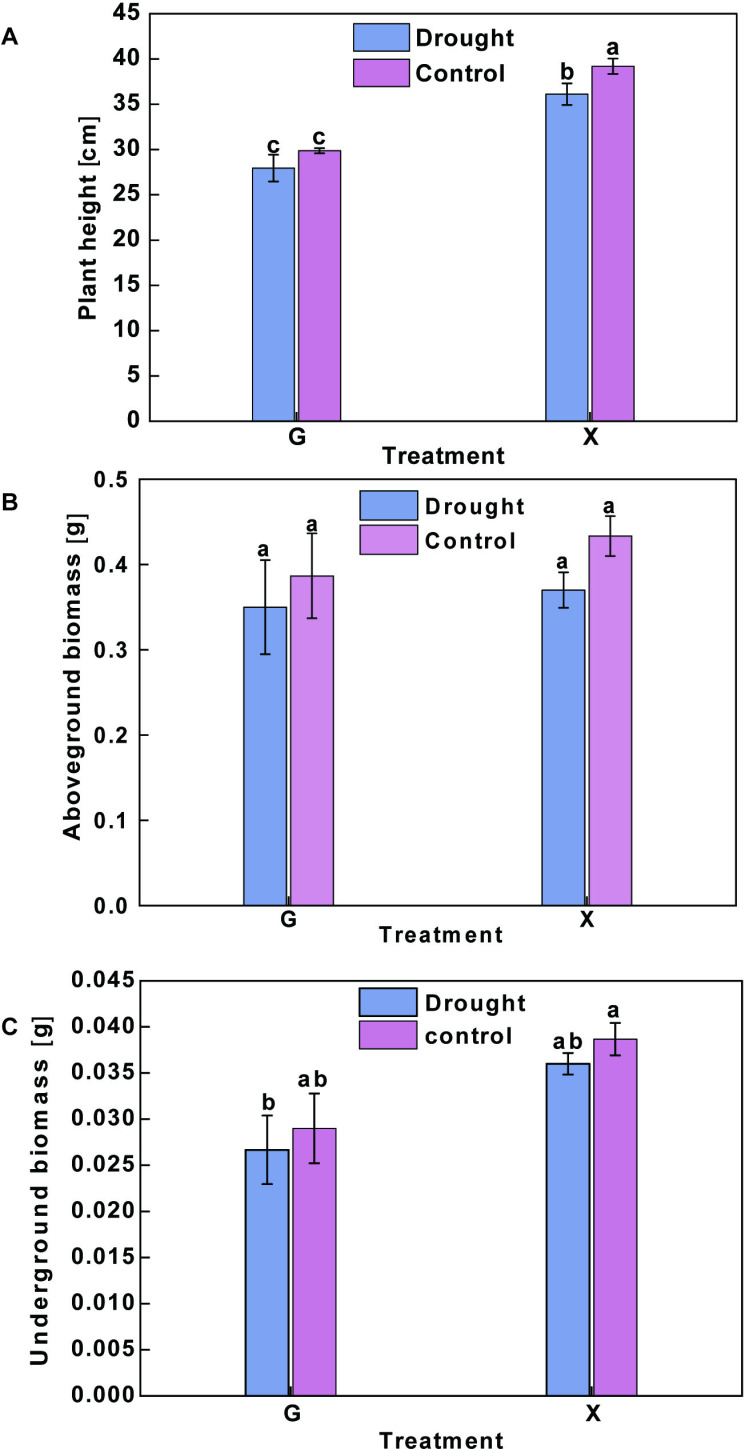
Changes in the growth of two oat varieties under drought stress and normal water supply treatments. **A** Plant height. **B** Aboveground biomass. **C** Underground biomass. Different lowercase letters above the columns indicate significant differences (*p* < 0.05) between drought stress and normal water supply treatments based on ANOVA.

Different drought-tolerant oat varieties show varied growth responses to drought stress (S1 Table). Under drought stress, GD and XD displayed different degrees of reduction in plant height. No significant difference was observed between GD and GW, while the plant height of XD was significantly reduced (by 7.9%) compared to XW (*p* < 0.05) (Fig 1A). Additionally, the aboveground biomass of GD was 9.5% lower than that of GW, while the aboveground biomass of XD was 14.6% lower than that of XW, with no significant differences between treatments within varieties (*p* > 0.05) (Fig 1B). Furthermore, the underground biomass of GD was 8.0% lower than that of GW, whereas the underground biomass of XD was 6.9% lower than that of XW, with no significant differences between treatments within varieties (*p* > 0.05) (Fig 1C). Variety G exhibited greater drought resistance than variety X.

G is forage oats *Avena sativa* cv,‘Grain King’, X is forage oats *Avena sativa* cv, ‘XiYue’.

### Effects of drought stress on the physiological indicators of oats

**Fig 2:**
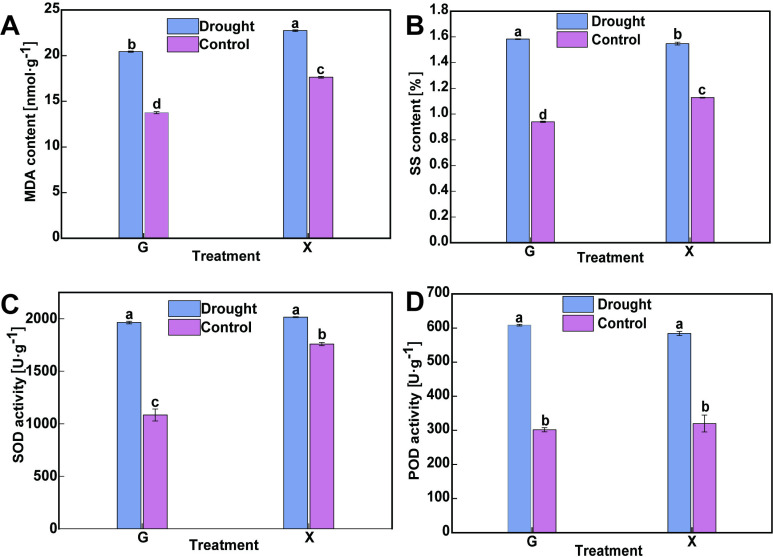
Changes in the physiological indexes of two oat varieties under drought stress and normal water supply treatments. **A** MDA content. **B** SS content. **C** SOD activity. **D** POD activity. Different lowercase letters above the columns indicate significant differences (*p* < 0.05) between drought stress and normal water supply treatments based on ANOVA. G is forage oats *Avena sativa* cv,‘Grain King’, X is forage oats *Avena sativa* cv, ‘XiYue’.

Several physiological indicators were measured after drought stress to further clarify the drought resistance of the two oat varieties (S2 Table). Under drought stress, the MDA content of GD was significantly higher (48.6%) than that of GW, while the MDA content of XD was significantly higher (28.9%) than that of XW (*p* < 0.05) (Fig 2A). The SS content of GD was significantly higher (by 68.5%) than that of GW, while it was significantly higher in XD (by 37.2%) than in XW (*p* < 0.05) (Fig 2B). The SOD activity of GD was significantly higher (by 81.3%) than that of GW, and that of XD was significantly higher (by 14.6%) than that of XW (*p* < 0.05) (Fig 2C). The POD activity of GD was significantly higher (by 101.7%) than that of GW, while it was significantly higher in XD (by 82.5%) than in XW (*p* < 0.05) (Fig 2D). This analysis suggests that drought stress affects the physiological indicators of oats, as revealed by the significant increase in osmoregulatory substances and antioxidant enzyme activities under drought stress. However, significant differences were observed between varieties under different treatments: variety G displayed a higher change margin in MDA, SS, SOD, and POD parameters and higher drought tolerance than variety X under drought stress conditions.

### Proteomics information identification

**Fig 3:**
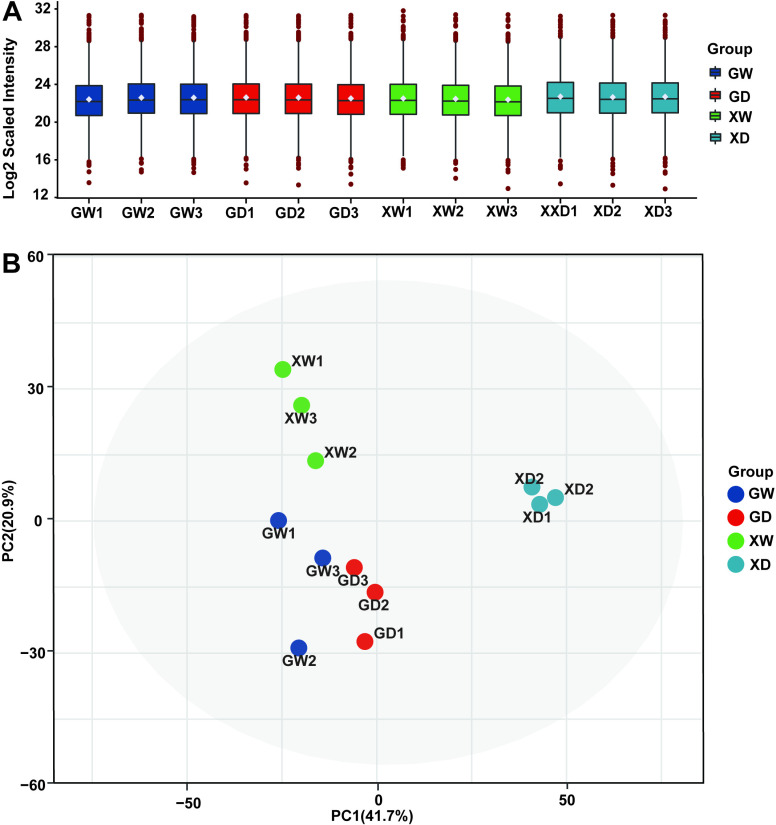
Normalized sample plots for each treatment for data quality control. **A** Normalized density line box plot. **B** Normalized principal component plot.

Seedling samples of the two oat varieties under different drought stress treatments were used, and the peptide spectral matching, unique peptide, protein groups, and quantified protein were determined as 17,690, 4,946, 1,821, and 1,817, respectively. Sample expression analysis and principal component analysis revealed that the 12 samples were classified into four main groups, demonstrating that the samples’ protein expression levels were biologically reproducible (Fig 3).

### DAP analysis

**Fig 4:**
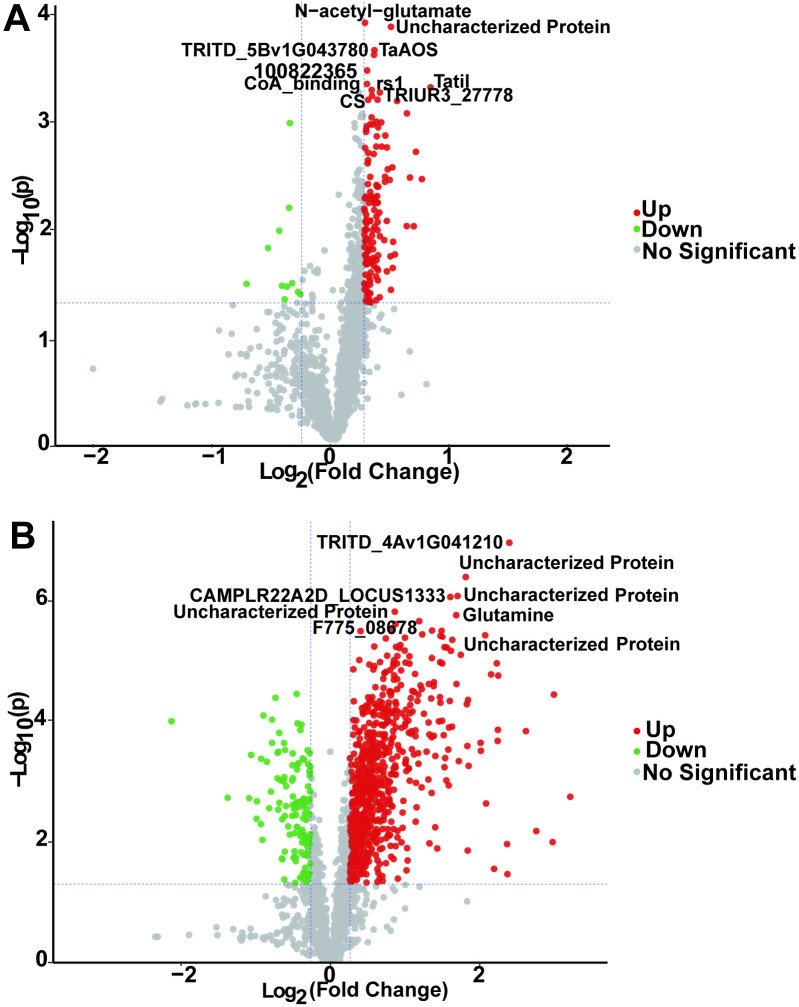
Volcano map of DAPs. **A** DAPs between GD and GW. **B** DAPs between XD and XW.

The quantitative analysis of proteins from the two drought-resistant varieties under different treatments revealed 846 DAPs (*p* < 0.05; cutoff at ratio fold-change > 1.2 or < 1/1.2), among which 724 were upregulated and 122 downregulated. GD had 140 upregulated and 11 downregulated DAPs compared to GW, while XD had 666 upregulated and 126 downregulated DAPs compared to XW. These results indicate remarkable differences in protein expression between the two oat varieties under drought stress conditions (Fig 4A, B).

Red and green indicate significantly upregulated and downregulated proteins, respectively.

### Identification of WGCNA modules related to drought resistance in oat varieties

**Fig 5:**
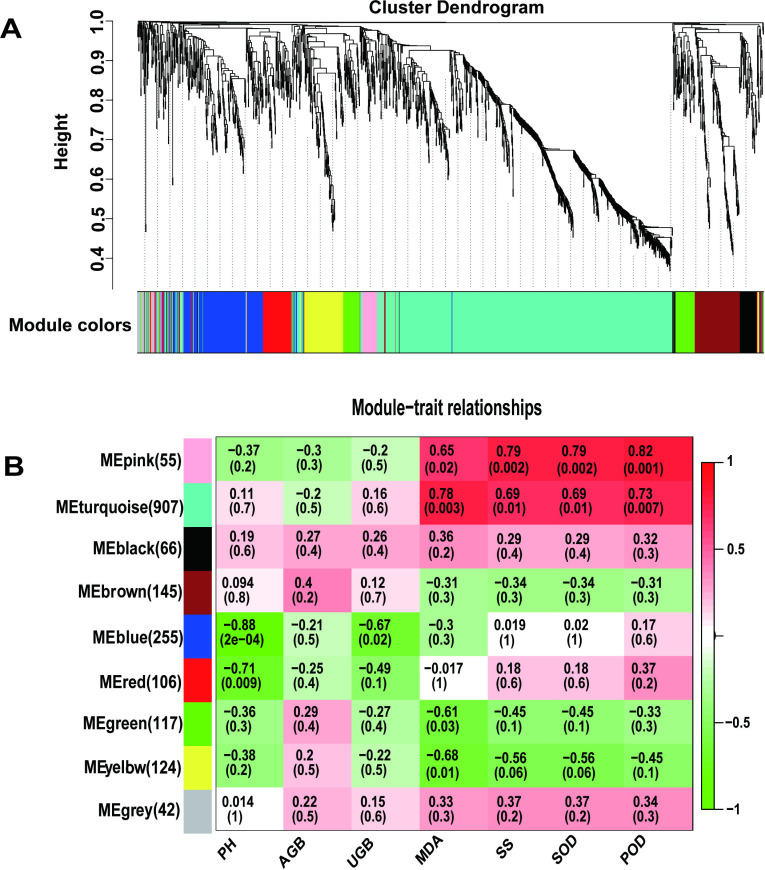
WGCNA of the sample characteristics and proteins of the two oat varieties under drought stress. **A** Stratified clustering of the nine WGCNA modules. **B** Correlation analysis of sample characteristics and proteins with changes in expression. Significant differences are indicated in parentheses, while correlation coefficients are indicated outside parentheses. PH, plant height; AGB, aboveground biomass; UGB, underground biomass.

Nine WGCNA modules were identified using WGCNA of sample characteristics and protein expression changes for the 1,817 quantified proteins (Fig 5). The results of the module-trait relationship indicated that 55 proteins in the “MEpink” module were highly correlated with POD (*r* = 0.82, *p* = 0.001) and SOD (*r* = 0.79, *p* = 0.002) activities and SS content (*r* = 0.79, *p* = 0.002), whereas they were moderately correlated with MDA content (*r* = 0.65, *p* = 0.02). The same proteins were not significantly correlated with plant height (*r* = −0.37, *p* = 0.2), aboveground biomass (*r* = −0.3 *p* = 0.3), and underground biomass (*r* = −0.2, *p* = 0.5). This observation may be due to a response threshold for drought stress in the phenotypic, physiological, and molecular aspects of plant tissues and organs. Within a certain threshold, drought stress only activates the stress response at the physiological and molecular levels of oats, and the effects on morphological and growth traits are not particularly evident.

In the MEpink module, the drought-resistant variety GD had 17 DAPs compared to GW, whereas the drought-susceptible variety XD had 5 DAPs compared to XW; the two varieties had two common DAPs, which were upregulated. These findings indicate that the 15 DAPs (excluding the two shared ones) in the MEpink module of the drought-tolerant variety may be essential in oat drought tolerance, and their synergistic expression can improve drought tolerance.

### GO enrichment analysis

**Fig 6:**
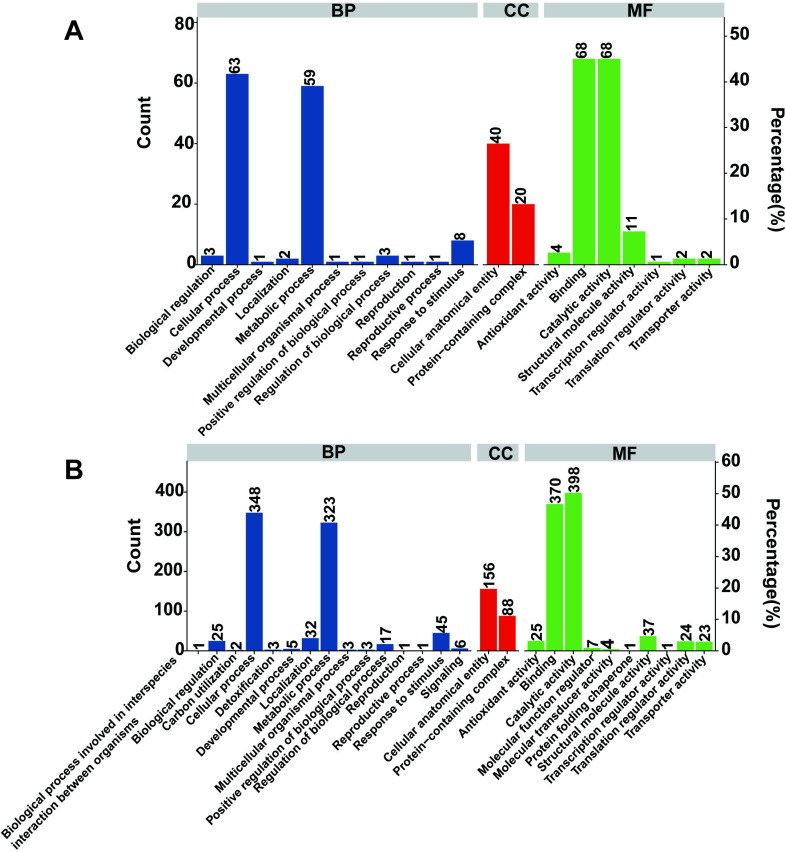
GO enrichment of DAPs, including BP, CC, and MF. **A** GO enrichment of DAPs between GD and GW. **B** GO enrichment of DAPs between XD and XW.

The GO enrichment analysis of DAPs in the leaves of the two oat varieties indicated that all DAPs were enriched in biological process (BP), cellular component (CC), and molecular function (MF). Specific proteins with different abundances are presented in Fig 6. In variety G (Fig 6A), BP was mainly enriched in the following GO terms: “cellular process,” “metabolic process,” “response to stimulus,” “regulation of biological processes,” “biological regulation,” and “localization” (and 11 other aspects). Additionally, CC was enriched in “anatomical entity” and “protein-containing complex.” Furthermore, MF mainly included the terms “catalytic activity,” “binding,” “structural molecule activity,” and “antioxidant activity.” In variety X (Fig 6B), BP was mainly enriched in “cellular process,” “metabolic process,” “response to stimulus,” “localization,” “biological regulation,” and “regulation of biological processes.” In CC, only two terms were enriched: “anatomical entity” and “protein-containing complex.” Furthermore, the DAPs in MF primarily had 10 functions, including “catalytic activity,” “binding,” “structural molecule activity,” “antioxidant activity,” “translation regulator activity,” and “transporter activity.” However, the 20 DAPs of the MEpink module were not significantly enriched in the GO database. The above results indicate that in terms of CC, MF, and BP, DAP enrichment levels were markedly different between the two oat varieties under drought stress conditions, revealing large differences in the protein levels of the two varieties in response to drought stress.

### KEGG pathway enrichment analysis

**Fig 7:**
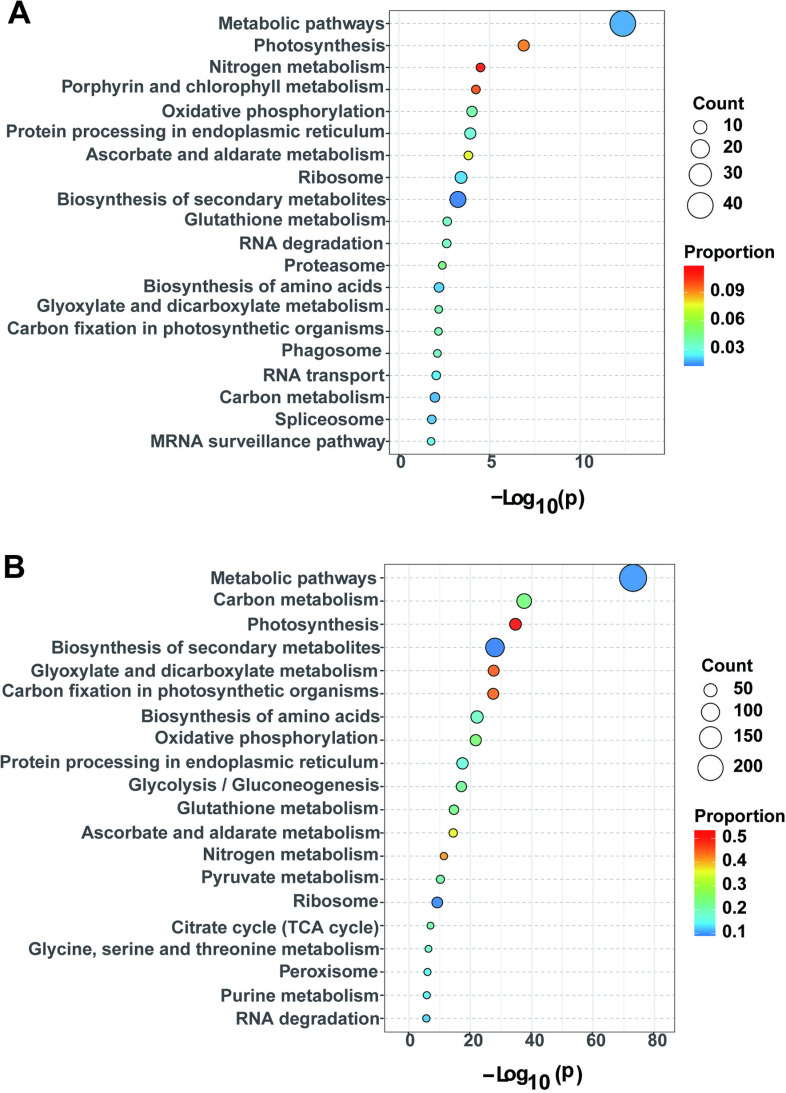
KEGG pathway enrichment bubble map of the top 20 DAPs. A KEGG pathway enrichment map of the top 20 DAPs between GD and GW. B KEGG pathway enrichment map of the top 20 DAPs between XD and XW. The vertical coordinate indicates the enriched pathway, while the horizontal coordinate indicates the negative logarithmic transformation of the *p*-value. *p* < 0.05 indicates that the function is significantly enriched, with smaller *p*-values indicating significantly higher functional enrichment. The color of the circle denotes the rich factor: Rich factor = (a/b)/(c/d), where a is the number of DAPs annotated to the term, b is the total number of DAPs annotated to the term, c is the number of background proteins of the term, and d is the total number of background proteins of the term. A larger Rich factor indicates a higher level of enrichment, and the size of the circle indicates the number of differentially abundant proteins enriched in the pathway.

The KEGG pathway enrichment analysis revealed that DAPs were enriched in 46 and 97 KEGG metabolic pathways in the G and X varieties, respectively, under drought stress. In variety G (Fig 7A), DAPs were significantly enriched in 27 KEGG pathways under drought stress (*p* < 0.05), including metabolic pathways, photosynthesis, nitrogen metabolism, porphyrin and chlorophyll metabolism, oxidative phosphorylation, and protein processing in the endoplasmic reticulum. In variety X (Fig 7B), DAPs were significantly enriched in 51 KEGG pathways under drought stress (*p* < 0.05), including metabolic pathways, carbon metabolism, photosynthesis, biosynthesis of secondary metabolites, glyoxylate and dicarboxylate metabolism, and carbon fixation by photosynthetic organisms.

The KEGG enrichment analysis of the 20 DAPs in the MEpink module indicated enrichment in 12 and 3 KEGG metabolic pathways in the G and X varieties, respectively, under drought stress. Among them, DAPs of variety G were significantly enriched in five KEGG pathways (*p* < 0.05), including the mRNA surveillance pathway, ribosome, phenylalanine metabolism, photosynthesis, and phagosome, whereas DAPs of variety X were significantly enriched in three KEGG pathways (*p* < 0.05), including photosynthesis, phagosome, and plant-pathogen interactions. Thus, the KEGG enrichment pathways of DAPs differed between the two oat varieties, implying that they may involve metabolic pathways in response to drought stress.

### Protein division in the MEpink module

In the MEpink module, 17 DAPs were identified in variety G under drought stress. All were upregulated, except for two DAPs that were shared with variety X (a chaperone protein DnaJ [ID: M8A0F5] and an uncharacterized protein [ID: A0A446J984]). The remaining DAPs were mostly involved in energy metabolism, protein translation, RNA processing, and amino acid metabolism: a 9-kDa polypeptide (ID: A0A0Q3IET4), carbamoyl-phosphate synthase (ID: Q9AXS0), temperature-induced lipid (TIL) transport protein (ID: I1IBR5), photosystem I subunit VII (ID: A0A446S4S2), PAP_fibrillin domain-containing protein (ID: A0A446MFG9), SpoU_sub_bind domain-containing protein (ID: A0A0Q3FXR3), SRP54 domain-containing protein (ID: A0A446KTE5), isoleucyl-tRNA synthetase (ID: I1H766), and seven uncharacterized proteins (ID: A0A446SZM6, A0A453AN53, A0A510B3S2, A0A446QWZ0, M5BPX4, A0A3B6FW66, and A0A453N829). Variety X had three DAPs (excluding the two shared with variety G) under drought stress, all of which were upregulated: peptide chain release factor (ID: M8CEY9) and two uncharacterized proteins (ID: A0A3B5YS83 and A0A3B6A287) primarily involved in RNA processing, protein stabilization, plant photosynthesis, and intracellular signal transduction.

## Discussion

### Common pathways to cope with drought stress in different oat varieties

Different oat varieties of the same species exhibit common drought tolerance pathways at the protein level. One such pathway involves the DnaJ protein, a heat stress protein primarily associated with protein folding, translocation, secretion, localization, and the reversion and degradation of denatured proteins. It plays a crucial role in maintaining intracellular protein homeostasis under adverse conditions and stabilizing protein complexes [30]. Studies on DnaJ protein expression in pepper placenta have shown that it may participate in the plant’s response to abiotic stress during the biosynthesis of stimulatory compounds [31]. Wang’s investigation into the function of the tomato (*Lycopersicon esculentum*) chloroplast-targeted DnaJ protein (LeCDJ2) and its ectopic expression in transgenic tobacco revealed that salicylic acid, drought, and pathogen attacks induced LeCDJ2 expression. Furthermore, the ectopic expression of LeCDJ2 in transgenic tobacco reduced the production of superoxide anion radicals (O_2_−) and hydrogen peroxide (H_2_O_2_) under drought stress. The overexpression of the tomato chloroplast-targeted DnaJ protein also improved the drought and cyanobacterial resistance of transgenic tobacco [32]. In the present study, DnaJ protein expression was significantly upregulated in both oat varieties, with higher expression observed in the drought-susceptible variety compared to the drought-resistant one. This finding suggests that the drought-susceptible variety may activate its response mechanisms to water deficit earlier than the drought-resistant variety, consistent with previous reports [33,34].

### Different pathways to cope with drought stress in different oat varieties.

Photosystem I (PSI) is a multisubunit membrane complex that catalyzes electron transfer involving iron redox proteins, which bind to fumarate and nitrate reduction and reduced nicotinamide adenine dinucleotide phosphate (NADPH). In higher plants, the PSI complex includes 4–80 kDa polypeptides in addition to light-harvesting complexes I (LHCI), among which the 9-kDa polypeptide subunit is a protease that may play a functional role under unfavorable growth conditions [35]. In this study, the expression of the 9-kDa polypeptide subunit was significantly upregulated in the strongly drought-resistant variety G, whereas it remained insignificant in the drought-susceptible variety X. This finding suggests that strongly drought-tolerant oat varieties may respond to drought stress by enhancing energy production during cellular photosynthesis, thereby reestablishing homeostasis and complex energy metabolic pathways. This observation aligns with previous research showing that the expression of photosynthesis-related proteins is upregulated in drought-resistant maize varieties [36].

Arginine serves as a precursor for the production of compounds that act as second messengers, such as nitric oxide (NO) and polyamines (including spermidine and putrescine), which play crucial roles in regulating stress resistance in plants [37–40]. For instance, exogenous chitosan and spermine have been shown to mitigate drought-induced oxidative damage and enhance the content of total phenols and flavonoids in white clover (*Trifolium repens* L.) [40]. The carbamoyl-phosphate synthase subunit is a catalytic enzyme essential for converting ornithine to citrulline during arginine biosynthesis and plays a role in plant resistance mechanisms [41]. This observation was further supported by the findings of the present study, where the enzyme was significantly upregulated under drought stress in the drought-resistant variety. Its activity catalyzed the synthesis of ornithine into citrulline, releasing energy to sustain plant growth under adverse conditions [40]. Conversely, this enzyme was not significantly upregulated in the drought-susceptible variety.

Lipoproteins are critical enzymes of the lutein cycle and play a key role in protecting the photosynthetic apparatus from oxidative damage caused by excessive light exposure [42]. Two homologous cDNAs from wheat (*TaTIL*) and Arabidopsis (*AtTIL*) encode lipoproteins containing three structurally conserved regions that potentially serve as ligands with diverse structures and functions for the synthesis of phytosteroid hormones, such as 2,4-epi-oligolactone, which enhance plant tolerance to heat and cold stress [43,44]. AtTIL transport proteins are also involved in regulating plant tolerance to oxidative stress [45], with plant TIL transport proteins counteracting severe heat stress-induced lipid peroxidation [46]. Additionally, *AtTIL*, a lipid-like transport protein gene, is implicated in plant salt tolerance [47]. However, the role of TIL transport proteins in drought tolerance in plants has not been previously reported. In this study, TIL transport proteins were significantly upregulated in the drought-tolerant variety under drought stress, whereas their relative abundance did not change remarkably in the drought-susceptible variety. This finding suggests that TIL transport proteins may play a role in regulating plant drought tolerance and improving drought resistance.

Plastoglobuli, lipoprotein particles ranging in diameter from 30 nm to 5 μm, are present in plastids [48]. A family of fibrillin (FBN) proteins has been identified within these lipoproteins. The *FBN* gene is induced by various stresses, including high temperature, low temperature, drought, salt, herbicides, and strong light, mediating ABA-related resistance signaling and regulating the synthesis of jasmonic acid, triacylglycerol, and plastoquinone [49–52]. In this study, the expression of the PAP_fibrillin domain-containing protein in variety G was significantly upregulated under drought stress, consistent with the increased expression of FBN proteins under drought stress observed in Arabidopsis, tomato, potato, tobacco, and other plants [49,53,54]. This finding suggests that the PAP_fibrillin domain-containing protein may play a role in signal transduction and response to drought stress in oat chloroplasts. Further research is necessary to determine whether this protein has a critical role in recognizing and responding to drought stress and to elucidate its specific functions.

Serine/arginine-rich proteins are primarily involved in the assembly and splicing of eukaryotic mRNA precursors. They are critical components of the mRNA spliceosome, playing a unique regulatory role in splicing mechanisms [55]. Plants respond to stress by regulating splicing factors; for instance, variable splicing of the *OsWRKY45* allele is involved in the drought stress response [56], and the overexpression of *ScMYBAS1–2* and *ScMYBAS1–3* splice transcripts promotes changes in plant growth under water deficiency and drought conditions [57]. In this study, the SRP54 domain-containing protein was significantly upregulated under drought stress in variety G, but not in variety X. This finding suggests that the SRP54 domain-containing protein may play a critical role in mediating the oat response to drought stress as a splicing factor.

In the MEpink module, seven uncharacterized proteins were significantly upregulated under drought stress in variety G but not in variety X. These proteins, with unknown structures and functions, may play a crucial role in the regulatory network of drought stress and contribute to improving drought resistance in oats. Further studies on these uncharacterized proteins are needed to elucidate the molecular mechanisms underlying the oat response to drought stress.

## Conclusions

**Fig 8:**
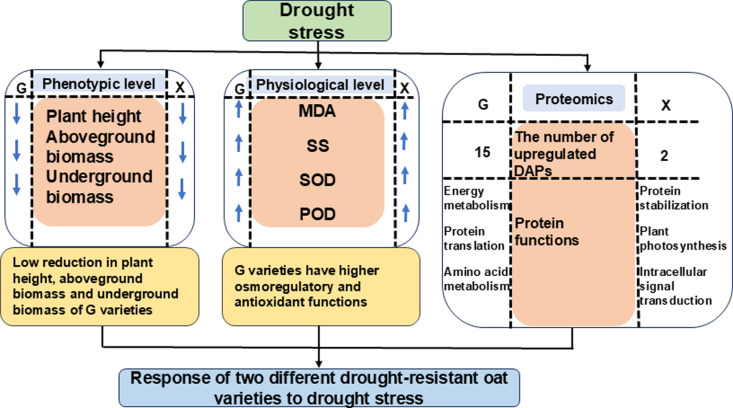
Different responses of two oat varieties to drought stress.

Our study investigated the mechanisms of drought tolerance in drought-resistant (Grain King) and drought-susceptible (XiYue) oat varieties from phenotypic, physiological, and proteomic perspectives (Fig. 8). Phenotypic and physiological data demonstrated that the drought-resistant oat variety outperformed the drought-susceptible variety under drought stress. Proteomic analysis suggested that the high drought resistance of Grain King is likely associated with the upregulation of proteins, including the 9-kDa polypeptide, the subunit of carbamoyl-phosphate synthase, temperature-induced lipid transport protein, PAP fibrillin domain-containing protein, and SRP54 domain-containing protein. This study elucidates the relationships between physiological and proteomic factors contributing to drought resistance and advances our understanding of drought tolerance mechanisms in different oat varieties.

## Supporting information

S1 Table Raw data of fig 1
(DOCX)

S2 Table Raw data of fig 2.
(DOCX)
