# Supplementary material for: Proteomic analysis of oat (Avena sativa L.) under drought stress using tandem mass tag labeling
Source: PLoS One. 2025 Apr 29;20(4):e0322022. doi: 10.1371/journal.pone.0322022 (PMC12040234; doi:10.1371/journal.pone.0322022)
Supplement: S1 Table — Raw data of fig 1 (DOCX) [file pone.0322022.s001.docx]

Table S1 Data of three phenotypic indicators

| Plant height | | | | Aboveground biomass | | | | Underground biomass | | | |
| --- | --- | --- | --- | --- | --- | --- | --- | --- | --- | --- | --- |
| G | | X | | G | | X | | G | | X | |
| D | W | D | W | D | W | D | W | D | W | D | W |
| 30.0 | 31.0 | 37.5 | 46.2 | 0.294 | 0.31 | 0.412 | 0.47 | 0.022 | 0.023 | 0.036 | 0.038 |
| 29.0 | 29.0 | 37.4 | 39.0 | 0.458 | 0.48 | 0.36 | 0.39 | 0.034 | 0.036 | 0.034 | 0.036 |
| 23.0 | 33.0 | 36.0 | 37.0 | 0.296 | 0.37 | 0.344 | 0.44 | 0.024 | 0.028 | 0.038 | 0.042 |
| 30.0 | 30.0 | 34.0 | 36.0 |  |  |  |  |  |  |  |  |
| 31.6 | 29.0 | 38.0 | 36.0 |  |  |  |  |  |  |  |  |
| 34.0 | 35.0 | 36.0 | 39.1 |  |  |  |  |  |  |  |  |
| 35.2 | 36.1 | 36.0 | 37.6 |  |  |  |  |  |  |  |  |
| 27.5 | 29.0 | 35.0 | 35.0 |  |  |  |  |  |  |  |  |
| 22.0 | 23.0 | 36.0 | 44.1 |  |  |  |  |  |  |  |  |
| 31.0 | 32.0 | 36.2 | 39.7 |  |  |  |  |  |  |  |  |
| 31.0 | 31.0 | 36.5 | 38.2 |  |  |  |  |  |  |  |  |
| 31.5 | 35.0 | 35.1 | 44.0 |  |  |  |  |  |  |  |  |
| 15.5 | 21.0 | 34.6 | 39.2 |  |  |  |  |  |  |  |  |
| 18.0 | 22.0 | 36.4 | 36.8 |  |  |  |  |  |  |  |  |
| 30.0 | 32.0 | 37.1 | 40.1 |  |  |  |  |  |  |  |  |

Note: Aboveground biomass and Underground biomass per weight are averages of 5 plants
