# Supplementary material for: Proteomic analysis of oat (Avena sativa L.) under drought stress using tandem mass tag labeling
Source: PLoS One. 2025 Apr 29;20(4):e0322022. doi: 10.1371/journal.pone.0322022 (PMC12040234; doi:10.1371/journal.pone.0322022)
Supplement: S2 Table — Raw data of fig 2. (DOCX) [file pone.0322022.s002.docx]

Table S2 Data of four physiological indicators

| SOD | | | | | | | | |
| --- | --- | --- | --- | --- | --- | --- | --- | --- |
|  | D | | | | W | | | |
|  | Ⅰ | Ⅱ | Ⅲ | avg. | Ⅰ | Ⅱ | Ⅲ | avg. |
| G | 1942.146 | 1983.863 | 1965.322 | 1963.777 | 968.972 | 1150.654 | 1130.467 | 1083.364 |
| X | 2007.039 | 2020.944 | 2016.309 | 2014.764 | 1729.346 | 1769.72 | 1776.449 | 1758.505 |
| POD | | | | | | | | |
|  | D | | | | W | | | |
|  | Ⅰ | Ⅱ | Ⅲ | avg. | Ⅰ | Ⅱ | Ⅲ | avg. |
| G | 613 | 606 | 606 | 608.3 | 290 | 305 | 310 | 301.7 |
| X | 572 | 592 | 588 | 584.0 | 345 | 345 | 270 | 320.0 |
| MDA | | | | | | | | |
|  | D | | | | W | | | |
|  | Ⅰ | Ⅱ | Ⅲ | avg. | Ⅰ | Ⅱ | Ⅲ | avg. |
| G | 20.382 | 20.477 | 20.466 | 20.44 | 13.691 | 13.971 | 13.612 | 13.758 |
| X | 22.560 | 22.840 | 22.807 | 22.74 | 17.642 | 17.477 | 17.791 | 17.637 |
| SS connect | | | | | | | | |
|  | D | | | | W | | | |
|  | Ⅰ | Ⅱ | Ⅲ | avg. | Ⅰ | Ⅱ | Ⅲ | avg. |
| G | 1.583 | 1.588 | 1.578 | 1.58 | 0.948 | 0.933 | 0.938 | 0.94 |
| X | 1.568 | 1.543 | 1.533 | 1.55 | 1.128 | 1.123 | 1.133 | 1.13 |
